# Factors associated with student success in an introductory level animal science course

**DOI:** 10.1093/tas/txag091

**Published:** 2026-07-02

**Authors:** M Tipton, J Ahola, Q Scott, C Cramer

**Affiliations:** Department of Animal Sciences, Colorado State University, Fort Collins, CO 80521, United States; Department of Animal Sciences, Colorado State University, Fort Collins, CO 80521, United States; College of Agricultural Sciences, Colorado State University, Fort Collins, CO 80521, United States; Department of Animal Sciences, Colorado State University, Fort Collins, CO 80521, United States

**Keywords:** animal science course, higher education, student success, student support

## Abstract

The impact of demographic factors and student academic behaviors on student success are well-documented in many science disciplines, yet little data exists for animal science programs. The objective of this study was to determine if academic behaviors or demographic factors were associated with students’ final grades in an introductory animal science course at Colorado State University. Students enrolled during the fall 2021, 2022, and 2023 semesters were anonymously surveyed at semester’s end regarding their final grade, academic behaviors, and demographics. Separate regression models assessed associations between final course grade and extracurricular involvement, perceived adequacy of studying/notetaking strategies, attendance patterns, high school GPA (HSGPA), race, number of times enrolled in the course, and first-generation status (FG). Students that connected with faculty and those that were involved in clubs, research, or voluntary animal feeding were associated with a lower likelihood of failing (earning a grade of D or F) compared to those who were not (*P* = 0.006, *P* = 0.003, *P* = 0.013, *P* = 0.001, respectively). Students with perceived inadequacies in studying or notetaking strategies were associated with a higher likelihood of failing compared to those who felt their skills were adequate (*P* = 0.002 and *P* < 0.001, respectively), yet no differences were seen between students with different notetaking strategies (*P* = 0.662) or quantities of time spent studying for quizzes (*P* = 0.435) or exams (*P* = 0.370). Students who attended 80% to 90% and < 70% of class periods were associated with a higher likelihood of failing, compared to those who attended 100% of class periods (*P* = 0.010 and *P* = 0.005, respectively), but no differences were seen between those attending 100% of classes and those attending 90% to 100% of them (*P* = 0.185), nor between those attending 80% to 90% of classes and those attending < 70% of them (*P* = 0.203). An interaction occurred between FG and HSGPA (*P* = 0.040), showing that the effect of FG on likelihood of failing differs by HSGPA category. Racially minoritized students and students retaking the course had a higher probability of failing the course than their respective counterparts (*P* < 0.001 and *P* = 0.003, respectively). This study highlights the roles of involvement, attendance, perceived inadequacies in learning behaviors, and various demographics in academic performance and can be used to inform future student support programs in animal science disciplines.

## Teaser

What factors impact the success of first-year students in an animal science degree program? How do these compare with students in other science disciplines?

## Graphical Abstract

**Graphical Abstract:**
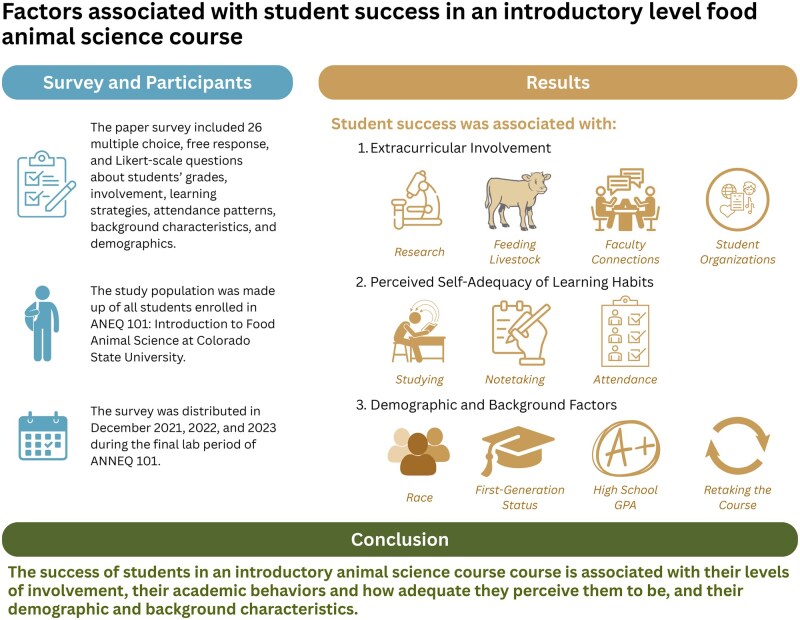

## Introduction

In higher education institutions across the United States, science, technology, engineering, and mathematics (STEM) disciplines are experiencing enrollment rates that are higher than ever, with shifts towards increased racial and gender diversity (Soler 2025). An uptick in non-traditional student enrollment has been noted as well, including increased enrollment of students returning to school after enlisting in the military, students returning after taking some time off to work or start a family, and students only taking courses part-time. Animal science is one such STEM discipline that is experiencing these trends in higher education, with over 8000 Animal Science degrees awarded nationwide in 2022, compared to just 6000 in 2012, as well as a shift from a primarily white male dominated demographic to a primarily female dominated demographic with a more diverse group of racial and ethnic identities represented in the last decade (Data USA 2023). Additionally, enrollment in animal science programs is most popular in institutions with affiliated veterinary schools and institutions that serve as a land-grant university. Of the top ten institutions with the highest proportion of degrees awarded in animal sciences, 90% have an affiliated AVMA-accredited veterinary school and 80% serve as their state’s land-grant institution (Data USA 2023; AVMA 2025). One potential reason for preference for enrollment in these institutions could be the high proportion of students that enter the discipline to pursue a career in veterinary medicine or to enter production livestock industries, both of which are supported by and often work with land-grant universities.

Colorado State University (CSU), which is both affiliated with an accredited veterinary school and serves as Colorado’s land-grant university, has experienced a similar rise in animal science degree enrollment and expansion of racial-, ethnic-, and gender-based diversity within the degree program. The university reports a nearly 20% increase in total animal science undergraduate enrollment and a 60% increase in racially minoritized student enrollment in the past decade (Colorado State University 2025a). Further, 88% of students enrolled in the animal sciences degree program at CSU identify as female, making it a female-dominated major, unlike many other STEM disciplines (Colorado State University 2025a). However, CSU is experiencing significantly lower 4- and 6-year graduation rates for animal science students when compared to peer institutions offering the same major. For example, the current 4- and 6-year graduation rates at CSU are 51.9% and 66.7% respectively, which is much lower than the 62% 4-year graduation rate for the entire State University System of Florida, the 84.2% 6-year graduation rate of Texas A&M, and the 88% 6-year graduation rate of the University of Illinois Urbana-Champaign (University of Illinois Urbana Champagne n.d.; Page 2024; Texas A&M University 2024; Colorado State University 2025a). While these universities have more selective admissions criteria and therefore lower acceptance rates compared to CSU (College Board 2025; Colorado State University IRPE 2025; University of Illinois Urbana Champagne n.d.), which may impact graduation rates, they are all quite similar to CSU in that they are large, land-grant universities with heavy focuses on agricultural science disciplines. Moreover, when comparing graduation rates across departments university-wide, the Department of Animal Sciences has 4- and 6-year graduation rates of 40.1% and 58.1% respectively, which are lower than those of more than half of the other departments at CSU, as well as lower than the 51.9% 4-year and 66.7% 6-year average graduation rates for all STEM departments combined at CSU (Colorado State University 2025a). Graduation rates are one of several metrics used to measure student success, alongside outcomes such as skills, grade point average, self-efficacy, holistic growth, self-directed learning, and post-graduation career status. General student success indicators are difficult to track across an entire university. Therefore, graduation rates serve as a simple but limited system-wide indicator. Further, low graduation rates often signal broader challenges with student success. Rising enrollment and increased diversity at CSU, coupled with persistently low graduation rates, raise concerns about whether existing support structures are truly meeting student needs, which may negatively impact future enrollment and recruitment to the university. These shifting demographics underscore the importance of ensuring support systems are both effective and equitable enough for students of all identities and backgrounds to succeed.

Improving academic outcomes among students requires an understanding of how students’ academic behaviors, activities, and attitudes contribute to their academic performance. Research shows that involvement in campus clubs, organizations, and educationally purposeful activities improves grades, retention, and application of learning (Kuh et al. 2008; Hawkins 2015). Additionally, students engaged in undergraduate research achieve higher GPAs and graduation rates compared to those that are not (Schneider et al. 2021). Such involvement also enhances belonging and authenticity, which benefits performance, motivation, and perceived support (Freeman et al. 2007; Cwik and Singh 2022; Verbree et al. 2025). Involvement also fosters meaningful connections with faculty and staff, which increases persistence within a major and an institution (Hawkins 2015; Xu 2018). Additionally, students are more likely to utilize existing academic and social support structures when they are promoted by trusted peers or faculty members, further reinforcing the value of creating meaningful connections with faculty and other students alike (Kearns et al. 2015; Qayyum 2018; Hagler et al. 2023). Beyond involvement, effective learning strategies such as improved notetaking, studying methods and hands-on, experiential, and collaborative learning approaches are linked to higher test scores, greater interest, and stronger self-efficacy (Mendezabal 2013; Sebesta and Speth 2017; Bradberry and De Maio 2018; Burch et al. 2019; Oje et al. 2021). Studies have also linked attendance patterns to student success outcomes (Al Hazaa et al. 2021), though this relationship may be influenced by other factors like in-class participation and demographic or background factors (Halpern 2007; Kim et al. 2020). Lastly, numerous studies have examined the role that demographic factors and background characteristics play in student success, and from these, it has been determined that race, gender, socioeconomic background, and first-generation college status can create hidden obstacles to academic success (Crisp et al. 2009; Whalen and Shelley 2010; Çiftçi and Cin 2017; Zuo et al. 2017). Overall, involvement, strong academic and social connections, effective learning strategies, consistent attendance, and numerous demographic and background factors all play major roles in student success in higher education. These factors could be used to inform current and future student support systems and topics to improve university student success on a more holistic level.

To develop effective support systems more tailored for animal science students, however, it is essential to understand which factors specifically influence success within the discipline. Limited studies have highlighted benefits of hands-on learning and critical thinking in animal science courses (Ferree et al. 2022; Ragland et al. 2023; Scott et al. 2023), as well as self-motivation (Lavoie 2019), positive attendance patterns (McMillan et al. 2009), and participation in learning communities (Zinn et al. 2015). Most other research has focused on demographic and background characteristics (Hale and Rosenkrans 2004; Gwazdauskas et al. 2014; White et al. 2015; Vinyard et al. 2022), yet even these findings are in conflict regarding their impact on student success. For example, Gwazdauskas et al. (2014) found gender to have a significant effect on final course grade in an Animal Anatomy and Physiology course whereas Vinyard et al. (2022) found no effect of gender on academic performance. More work is therefore needed to identify academic behaviors, activities, and attitudes associated with success in animal sciences, as well as to clarify how demographic and background factors influence outcomes. The objectives of this study are to (1) determine which academic behaviors (e.g., involvement, attendance patterns, notetaking strategies, and study practices strategies) were positively or negatively associated with final grades in an introductory animal science course, and (2) to identify which demographic and background characteristics of animal science students were positively or negatively associated with final grades in the same course.

## Methods

Data collection for this retrospective observational study took place at Colorado State University (CSU) in December 2021, 2022, and 2023 during the final laboratory meeting of the course titled ANEQ 101: Food Animal Science. The CSU Institutional Review Board (5789) approved the research plan prior to data cleaning and analysis.

### ANEQ 101 course overview

In the Department of Animal Sciences at CSU, the “Food Animal Science” (ANEQ 101) course is required for all students within the animal science major and is often taken during their first semester. It also serves as a common elective course for other students in the College of Agricultural Sciences, those interested in veterinary medicine, and other students at the university. The ANEQ 101 course objectives are to (1) Explore the development, organization, trends and management of all sectors of the livestock industry and (2) Apply scientific principles to the production of food and fiber. The course includes 3, 50-minute lectures and a required 110-minute lab period per week. During the lecture portion, students are provided with material on all sectors of the livestock industry, as well as basic livestock disciplines such as anatomy, reproductive physiology, nutrition, genetics, handling and husbandry, and veterinary care. Students also receive guest lectures from faculty members in the department and monthly club/activity announcements to keep them informed about what is happening within the department and ways to get involved. Though no points are awarded specifically for attendance, attendance is listed as required for both lecture and laboratory sections, as many of the course assignments can only be completed to the fullest extent through in-person involvement. One highlight of the course is the opportunity for students to work hands-on with non-replacement dairy calves for the first 5 weeks of the semester. In those weeks, groups of 3–5 students are assigned to a week-old calf and tasked with learning how to feed, handle, weigh, and provide basic husbandry for their assigned calf. All students can volunteer as often as they wish to help during the twice-daily feedings and health checks for these calves. Volunteering to help feed and care for these calves is not required, nor is it attached to their final grade in any way. However, it can provide an opportunity for further hands-on and experiential learning outside of the course setting, as well as an opportunity for extracurricular involvement and more meaningful connections with faculty and teaching assistants. The course was chosen for analysis because it enrolls many first-semester students, a critical period for shaping long-term academic success (Tinto 2006). Additionally, according to internal data at Colorado State University, approximately 20–25% of students enrolled in this course over the last five years did not pass with a grade of C or higher, indicating the need for intervention.

### Study population and recruitment

The ANEQ 101 instructor developed a survey (included in supplementary material, available as supplementary data at *Translational Animal Science* online) to identify ways to support student success and improve the course’s curriculum and pedagogy in future semesters. The anonymous survey was administered during the final laboratory period of the course. The survey was not mandatory or associated with a student’s final grade. However, teaching assistants strongly encouraged students to complete the survey to help improve the course in future semesters. Only those that attended the final laboratory period were asked to complete the survey. No identifiers were collected during this process.

### Survey development and content

This survey included five sections: Course Overview, Course Performance, Weekly Labs, Challenges, and Demographics. In Sections 1 and 3, participants were asked multiple choice questions regarding overall perceptions of course material and its importance and relevancy, for both lecture (Section 1) and laboratory (Section 3) portions of the course. In Section 2, participants were asked to describe current grades, study habits, attendance patterns, and involvement in extracurricular activities related to animal sciences in a series of multiple choice and free-response questions. In Section 4, participants were asked Likert-scale questions with possible responses of “Strongly Agree,” “Agree,” “Disagree,” or “Strongly Disagree” regarding current challenges they faced in the course throughout the semester. Finally, Section 5 allowed respondents to describe their demographic identities via multiple choice and free-response questions. Not all survey questions were related to the current study objectives, so only 57.8% (*n* = 26/45) of questions were included in the final analysis. The questions that were used in the analysis, as well as the general categories they each fall under, are included in Supplementary Table 1, available as supplementary data at *Translational Animal Science* online.

### Statistical analysis

Survey responses were entered into Excel (Microsoft Corp., Redmond, WA) and stored for compilation and initial screening. All surveys were at least 70% complete, so all were included in final analysis. Data were cleaned and analyzed using Excel and SAS software (SAS Institute., Cary, NC). The outcome of interest was students’ self-reported final grade in ANEQ 101. Final course grade was initially analyzed as a categorical variable (A, B, C, D, or F) and later collapsed into a dichotomous categorical variable: “pass” (grades A, B, and C) and “fail” (grades D and F). Predictor variables included both categorical and dichotomous measures: lecture and laboratory attendance, high school GPA, year the survey was given, and gender were categorical, while extracurricular involvement, perceptions of note-taking and study habits, study difficulties, desire to improve study or note-taking habits, and demographic factors (first-generation status, number of course enrollments, transfer status, and race) were dichotomous (Table 1). Some variables were originally categorical as Likert-scale data (strongly agree/agree/disagree/strongly disagree) but were later collapsed to be dichotomous (agree/disagree; Table 1).

**Table 1:** List of all variables included in final analysis, including variable type and levels of each variable.

Variable category	Variable name	Variable type	Variable levels
**Outcome**	Final Grade	Binary	Pass (Grades A, B, and C)
			Fail (Grades D and F)
**Predictor—Involvement**	Research Involvement	Binary	Yes
			No
	Connecting with Faculty	Binary	Yes
			No
	Volunteering to Feed Calves	Binary	Yes
			No
	Club/Organization Involvement	Binary	Yes
			No
	Involvement (Comb.)	Binary	Yes (Involved in ≥ 1 Listed Activity)
			No (Not Involved in Any Listed Activities)
**Predictor—Attendance**	Lecture Attendance	Categorical	100% (Missed 0 Lectures)
			Almost 100% (Missed 1–2 Lectures)
			80% to 90% (Missed 3–10 Lectures)
			≤ 70% (Missed ≥ 11 Lectures)
	Laboratory Attendance	Categorical	100% (Missed 0 Labs)
			80% to 90% (Missed 1–3 Labs)
			≤ 70% (Missed ≥ 4 Labs)
**Predictor—Learning Strategies**	Perceived Adequacy of Notetaking	Binary	Yes(Agree/Strongly Agree)
			No (Disagree/Strongly Disagree)
	Perceived Adequacy of Study Habits	Binary	Yes (Agree/Strongly Agree)
			No (Disagree/Strongly Disagree)
	Experienced Difficulty Studying	Binary	Yes (Agree/Strongly Agree)
			No (Disagree/Strongly Disagree)
	Need to Improve Studying/Notetaking	Binary	Yes (Agree/Strongly Agree)
			No (Disagree/Strongly Disagree)
	Time Spent Studying	Continuous	Number of Minutes Spent Studying
	Notetaking Methods	Categorical	No Notes
			Write a Few Notes on Printed Slides
			Write Many Notes on Printed Slides
			Write Notes on Blank Paper
			Write Notes on a Tablet/Computer with the Slides
			Write Notes on Blank Tablet or Computer
**Predictor—Demographics and Background Factors**	High School GPA (HSGPA)	Categorical	≥ 4.00
			3.00–3.99
			< 3.00
	Race	Binary	White
			Racially Minoritized
	Gender	Categorical	Cis Male
			Cis Female
			Transgender
			Other/Prefer Not to Say
	Times Enrolled in ANEQ 101	Binary	First-Time Enrollee
			Returning Enrollee
	Transfer Student Status	Binary	Yes
			No
	First-Generation Student Status	Binary	Yes
			No
	Year Enrolled	Categorical	2021
			2022
			2023

Preliminary bivariate analyses (PROC FREQ) assessed associations between predictor variables (student involvement, learning strategies, perceived adequacy of learning strategies, attendance, year the survey was administered, and key demographic and background factors) and the outcome of interest (final course grade); Predictors with a *P-value* less than 0.20 were considered to have a conservative association with the outcome of interest and were thus retained for multivariable analyses (Dohoo et al. 2009). Chi-squared and *P*-values are reported for each bivariate analysis. To assess whether demographic factors acted as potential confounders in the relationship between other predictors and final grade, separate bivariate analyses were run for all combinations of demographic and predictor variables that showed conservative associations with the outcome (*p* < 0.20), including associations among demographic factors themselves. Demographic factors that were associated with another predictor (*p* < 0.05) were included as potential confounders in later multivariate analyses.

Nine multivariable models were constructed, one for each of the following primary predictors: overall involvement (all involvement-related predictors collapsed into one variable, yes/no), experienced difficulty while studying, perceived need to improve study skills, perceived inadequacy in time spent studying, perceived personal inadequacy of notetaking strategies, perceived need to improve notetaking strategies, lecture attendance, laboratory attendance, and demographic/background characteristics. Multivariable logistic regression (PROC GLIMMIX) in SAS (Version 9.4M8; SAS Institute Inc., Cary, NC), were used to assess if the predictors of interest were associated with the outcome, while controlling for potential confounders. All full multivariable models included high school GPA (HSGPA), race, first-generation (FG) student status, and times enrolled in the course, with the addition of the respective primary predictors (Table 2). Interactions were checked between the primary predictor of interest and each demographic factor included as a potential confounder in the model. Backwards stepwise removal was performed until only statistically significant variables and interactions (*p* < 0.05) remained in the models. The final models for the effects of involvement, attendance, and notetaking/studying habits on final course grade included the respective primary predictors for each model, HSGPA, race, and times enrolled in the course, while the final model for the effects of demographic factors on final course grade included race, times enrolled in the course, and the interaction between HSGPA and FG student status (Table 2). For each model, estimates, standard errors, and individual *P*-values were provided by the PROC GLIMMIX command against a referent level of each variable. Predictive probabilities and associated standard errors were also calculated for all levels of each variable included in the final models using the ilink command from the LSMEANS function of PROC GLIMMIX. These probabilities were calculated as the probability of a student earning a failing grade.

**Table 2:** List of all multivariable analysis models included in final analysis.

Model number	Predictor of interest	Full model	Final model
**1**	Involvement (Comb.)	Final Grade ∼ Involvement + HSGPA + Race + FG Status + Times Enrolled	Final Grade ∼ Involvement + HSGPA + Race + Times Enrolled
**2**	Experienced Difficulty While Studying	Final Grade ∼ Difficulty Studying + HSGPA + Race + FG Status + Times Enrolled	Final Grade ∼ Difficulty Studying + HSGPA + Race + Times Enrolled
**3**	Perceived Need to Improve Study Skills	Final Grade ∼ Perceived Need + HSGPA + Race + FG Status + Times Enrolled	Final Grade ∼ Perceived Need + HSGPA + Race + Times Enrolled
**4**	Perceived Inadequacy In Time Spent Studying	Final Grade ∼ Perceived Inadequacy + HSGPA + Race + FG Status + Times Enrolled	Final Grade ∼ Perceived Inadequacy + HSGPA + Race + Times Enrolled
**5**	Perceived Personal Inadequacy of Notetaking Strategies	Final Grade ∼ Perceived Inadequacy + HSGPA + Race + FG Status + Times Enrolled	Final Grade ∼ Perceived Inadequacy + HSGPA + Race + Times Enrolled
**6**	Perceived Need to Improve Notetaking Strategies	Final Grade ∼ Need to Improve Notes + HSGPA + Race + FG Status + Times Enrolled	Final Grade ∼ Need to Improve + HSGPA + Race + Times Enrolled
**7**	Lecture Attendance	Final Grade ∼ Lecture Attendance + HSGPA + Race + FG Status + Times Enrolled	Final Grade ∼ Lecture Attendance + HSGPA + Race + Times Enrolled
**8**	Laboratory Attendance	Final Grade ∼ Lab Attendance + HSGPA + Race + FG Status + Times Enrolled	Final Grade ∼ Lab Attendance + HSGPA + Race + Times Enrolled
**9**	Demographics and Background Characteristics	Final Grade ∼ HSGPA + Race + FG Status + Times Enrolled	Final Grade ∼ HSGPA*FG Status + Race + Times Enrolled

## Results

### Respondent demographics

Over the course of three consecutive fall semesters, 556 total survey responses were received (*n* = 174 in 2021, *n* = 184 in 2022, and *n* = 198 in 2023). Across all three semesters, the majority of respondents identified as female (77.0%; *n* = 428) and as white (64.6%; *n* = 359). Most of the respondents were in their first year of college (74.5%; *n* = 414) and nearly all were taking ANEQ 101 for the first time (96.2%). Most respondents were Animal Science majors (85.1%; *n* = 473) with most respondents (66.5%; *n* = 370) indicating an interest in attending veterinary school following graduation. Nearly a third (30.8%; *n* = 171) of respondents identified as a first-generation student in their family and 12.9% (*n* = 72) indicated being a transfer student. Across all three semesters, 7.4% (*n* = 41) of respondents indicated being enrolled in the University Honors Program and 34.0% (*n* = 189) indicated having a full- or part-time job while attending classes. Lastly, over half of the respondents (58.5%; *n* = 325) indicated originating from a state other than Colorado. Respondent demographics are described further in Table 3.

**Table 3:** Respondent demographics.

Demographic characteristic	*n*	Percentage
**Gender**		
** Female**	28	77.0%
** Male**	103	18.5%
** Transgender Female**	0	0.0%
** Transgender Male**	3	0.5%
** Gender Variant/Non-Conforming**	12	2.2%
** Prefer Not to Say**	5	0.9%
** Other**	2	0.4%
**Race**		
** American Indian or Alaska Native**	8	1.4%
** Asian**	22	4.0%
** Black or African American**	12	2.2%
** Hispanic**	83	14.9%
** White**	359	64.6%
** Multiracial**	65	11.7%
** Other**	3	0.5%
**Year in Undergraduate Program**		
** Year 1**	414	74.5%
** Year 2**	82	14.7%
** Year 3**	47	8.5%
** Year 4**	7	1.3%
**Year Enrolled**		
** 2021**	174	31.3%
** 2022**	184	33.1%
** 2023**	198	35.6%
**Major (At Start of Course)**		
** Animal Sciences**	492	88.5%
** Other College of Agricultural Sciences Major**	48	8.6%
** Non-College of Agricultural Sciences Major**	12	2.2%
**Major (At End of Course)**		
** Animal Sciences**	473	85.1%
** Other College of Agricultural Sciences Major**	57	10.3%
** Non-College of Agricultural Sciences Major**	21	3.8%
**High School GPA (HSGPA)**		
** ≥ 4.00**	197	35.69%
** 3.00–3.99**	335	60.69%
** < 3.00**	20	3.62%
**Additional Respondent Characteristics**		
** First Time Taking ANEQ 101**	535	96.2%
** Pre-Veterinary Interest**	370	66.5%
** Enrolled in University Honors College**	41	7.4%
** Transfer Student**	72	12.9%
** Out-of-State Student**	325	58.5%
** First-Generation (FG) Student**	171	31.32%

### Factors associated with final grade: bivariate analysis results

Involvement in the department, perceived adequacy of notetaking and study habits, and attendance patterns while enrolled in ANEQ 101 were all significantly associated with a student’s final course grade. More specifically, in regard to involvement within the department, connecting with faculty, being involved in department research, volunteering to feed dairy calves used for labs, and joining a club all were associated with a student’s final course grade (*p* < 0.02; Table 4). However, in the current study, only 23.0% (*n* = 128) of respondents indicated having connected one-on-one with a faculty member while being enrolled in the class, 6.12% (*n* = 34) of respondents indicated being involved in department research, 37.1% (*n* = 206) indicated volunteering to feed calves, and 40.6% (*n* = 226) indicated having joined a club. Further, when asked to select some reasons they might not have done as well in the course as they had hoped, 14.0% (*n* = 78) of respondents indicated the quality of their notetaking was inadequate, 41.5% (*n* = 231) indicated they did not study as much as they should have, and 39.4% (*n* = 219) indicated it was difficult for them to study and/or prepare for quizzes and exams. When the effects of learning strategies such as notetaking and study habits on final grade were analyzed in a bivariate analysis, an association was seen between final course grade and perceived adequacy of notetaking ability (*X^2^ *= 14.84; *p* < 0.0001; Table 4), perceived adequacy of the amount of time spent studying (*X^2^ *= 8.65; *p* = 0.003; Table 4), perceived adequacy of study habits and skills (*X^2^ *= 9.76; *p* = 0.002; Table 4), and perceived difficulty of studying/preparing for quizzes and exams (*X^2^ *= 44.20; *p* < 0.0001; Table 4). However, there was no significant relationship between the act of taking notes in class (yes/no), regardless of the style of notetaking, and final grade (*X^2^ *= 0.19; *p* = 0.66). Similarly, there was no significant relationship between the amount of time a student spent studying and their final grade (*X^2^ *= 65.74; *p* = 0.62; Table 4).

**Table 4:** Bivariate analysis of the effects of all predictors of interest on students’ final course grade in ANEQ 101.

Predictor	X^2^	*P-*Value
**Involvement**		
** Connecting 1-on-1 with Faculty**	7.56	0.0060
** Involvement in Department Research**	6.24	0.0125
** Volunteering to Feed Laboratory Calves**	10.33	0.0013
** Involvement in a Student Club or Organization**	9.02	0.0027
**Notetaking**		
** Taking Notes in Class**	0.19	0.6616
** Perceived Inadequacy in Personal Notetaking Ability**	14.84	< 0.0001
** Perceived Need to Improve Notetaking**	38.76	< 0.0001
**Study Habits**		
** Perceived Inadequacy in Time Spent Studying**	8.65	0.0033
** Experienced Difficulty in Studying/Preparing for Quizzes and Exams**	44.20	< 0.0001
** Perceived Need to Improve Study Skills/Habits**	9.76	0.0018
** Time Spent Studying for Quizzes**	2.73	0.4353
** Time Spent Studying for Exams**	4.27	0.3703
**Attendance**		
** Lecture Attendance**	26.13	< 0.0001
** Lab Attendance**	26.81	< 0.0001
**Demographics**		
** High School GPA**	13.20	< 0.0001
** Race**	35.08	< 0.0001
** Times Enrolled in the Course**	32.22	< 0.0001
** First-Generation Status**	10.31	0.0013
** Transfer-Student Status**	3.04	0.0790
** Gender**	9.52	0.1032

Additionally, across the three semesters of data collection, 20.8% (*n* = 116) of student respondents indicated attending all lectures, 37.6% (*n* = 209) indicated attending all but one or two lectures, 35.3% (*n* = 196) indicated all but 3–10 lectures, and 5.9% (*n* = 33) indicated missing more than 10 lectures. Similarly, 42.3% (*n* = 235) of student respondents indicated attending all labs, 53.2% (*n* = 296) indicated attending all but one or two labs, and 4.3% (*n* = 24) indicated missing three or more labs. From initial bivariate analyses, an association was seen between a student’s final grade and their lecture attendance (*X^2^ *= 26.1305; *p* < 0.0001; Table 4), as well as between final grade and their laboratory period attendance (*X^2^ *= 26.8053; *p* < 0.0001; Table 4).

Lastly, numerous demographic and background factors were tested via bivariate analysis to determine which ones were associated with final course grade. From this, it was determined that a student’s HSGPA (*X^2^ *= 13.1950; *p* < 0.0001; Table 4), the student’s race (*X^2^ *= 35.0766; *p* < 0.0001; Table 4), times enrolled in the course (*X^2^ *= 32.2150; *p* < 0.0001; Table 4), and whether a student was part of the first generation in their family to attend higher education (FG; *X^2^ *= 10.3071; *p* = 0.0013; Table 4) were all significantly associated with that student’s final course grade. Other demographic factors such as gender and transfer student status were not significantly associated with a student’s final course grade (*X^2^ *= 9.519, *p* = 0.1032; *X^2^ *= 3.0447; *p* = 0.079, respectively; Table 4). Bivariate analysis was also used to determine potential covariance between demographic factors and all other primary predictors, including other demographic factors. There were many significant associations between predictors and demographic factors, suggesting the presence of covariance; The results of this analysis are provided in Supplementary Table 5, available as supplementary data at *Translational Animal Science* online.

### Factors associated with final grade: multivariate analysis results

Based on results of the bivariate analysis, multivariable models were created for each of the following predictors: overall involvement (Model 1), experienced difficulty while studying (Model 2), perceived need to improve study skills (Model 3), perceived inadequacy in time spent studying (Model 4), perceived personal inadequacy of notetaking strategies (Model 5), perceived need to improve notetaking strategies (Model 6), lecture attendance (Model 7), laboratory attendance (Model 8), and demographic/background characteristics (Model 9) (Table 2).

When analyzing the effects of extracurricular involvement on final grade in multivariate analysis, responses for each of the involvement activities asked in the survey were combined to create a new binary variable for overall involvement, which indicated involvement in at least one activity (yes/no). Model 1 was used to analyze the effects of this new involvement variable on final grade, which showed that students who were involved in at least one extracurricular activity within the department had a 27.4% predicted probability of failing ANEQ 101, which is significantly lower than the 45.2% probability for those that were not involved in any way, when controlling for high school GPA (HSGPA), race, and number of times enrolled in the course. (*p* = 0.0055; Table 5).

**Table 5:** Multivariable analysis of the effects of student involvement on their probability of earning a failing grade in ANEQ 101.

Predictor	Estimate	*SE*	*P* value	Predicted Probability of Failing ± SE (%)
**Involvement**				
** Indicated Extracurricular Involvement**	Referent	–	–	27.41 ± 6.41%
** Did Not Indicate Extracurricular Involvement**	0.78	0.28	0.0055	45.21 ± 9.11%
**High School GPA**				
** ≥ 4.00**	Referent	–	–	15.82 ± 5.40%
** 3.00–3.99**	1.10	0.36	0.0024	36.04 ± 6.16%
** < 3.00**	2.17	0.60	0.0003	62.14 ± 13.34%
**Race**				
** White**	Referent	–	–	23.01 ± 6.38%
** Racially Minoritized**	1.25	0.27	< 0.0001	51.04 ± 8.19%
**Times Enrolled in ANEQ 101**				
** First-Time Enrollment**	Referent	–	–	19.27 ± 3.31%
** Not First-Time Enrollment**	1.70	0.52	0.0011	56.61 ± 13.18%

No significant interactions were observed, so main effects are reported for overall involvement (all involvement factors collapsed to indicate participation, or lack thereof, in at least one of the involvement factors) when controlling for HSGPA, race, and times enrolled in the course.

Models 2–6 were used to analyze the effects of studying and notetaking strategies on final grade when controlling for potential demographic variable confounders. For Model 2, which looked at the effect of a student experiencing difficulty when studying on final grade, it was determined that students that reported difficulty studying had a 45.5% probability of failing ANEQ 101 compared to the 16.7% probability for those who did not, when controlling for HSGPA, race, and times enrolled in the course (*p* < 0.0001; Table 6). However, Models 3 and 4 analyzed the effects of perceived needs to improve study skills and perceived inadequacies in time spent studying and found no effect of those predictors on final grade, when controlling for potential confounders (*p* = 0.9761 and *p* = 0.1369, respectively). When looking at the association between perceived personal inadequacy in notetaking strategies (Model 5), it was noted that students who indicated having this perceived inadequacy of their notetaking strategies were associated with a higher probability of failing compared to those that did not (45.2% and 26.5%, respectively; *p* = 0.0128; Table 7), when controlling for HSGPA, race, and times enrolled in the course. Lastly, estimates and predicted probabilities from Model 6 show that students who reported a need to improve their notetaking strategies had a 35.6% probability of earning a failing grade, which is significantly higher than the 10.4% probability of failing for those that did not feel the need to improve their notetaking strategies (*p* = 0.0006). Full outputs of this multivariate model are available in Supplementary Table 2, available as supplementary data at *Translational Animal Science* online. In short, results from Models 2–6 indicate that students that felt inadequate in their learning strategies had a higher probability of failing compared to those that did not.

**Table 6:** Multivariable analysis of the effects of experiencing difficulty studying for exams on a student’s probability of earning a failing grade in ANEQ 101.

Predictor	Estimate	*SE*	*P* value	Predicted Probability of Failing ± *SE* (%)
**Experienced Difficulty Studying**				
** Yes**	Referent	–	–	45.45 ± 8.68%
** No**	−1.42	0.30	< 0.0001	16.73 ± 5.24%
**High School GPA**				
** ≥ 4.00**	Referent	–	–	12.39 ± 4.49%
** 3.00–3.99**	1.06	0.36	0.0036	29.02 ± 5.80%
** < 3.00**	2.13	0.64	0.0009	54.23 ± 15.07%
**Race**				
** White**	Referent	–	–	18.25 ± 5.63%
** Racially Minoritized**	1.21	0.28	< 0.0001	42.85 ± 8.40%
**Times Enrolled in ANEQ 101**				
** First-Time Enrollment**	Referent	–	–	15.84 ± 2.98%
** Not First-Time Enrollment**	1.55	0.54	0.0043	47.08 ± 14.01%

No significant interactions were observed, so main effects are reported for having experienced difficulty when studying (yes/no), when controlling for HSGPA, race, and times enrolled in the course.

**Table 7:** Multivariable analysis of the effects of perceived personal inadequacy of notetaking strategies on a student’s probability of earning a failing grade in ANEQ 101.

Predictor	Estimate	*SE*	*P* value	Predicted probability of failing ± *SE* (%)
**Perceived Inadequacy of Notetaking**				
** Yes**	Referent	–	–	45.23 ± 9.98%
** No**	−0.83	0.33	0.0128	26.49 ± 6.35%
**High School GPA**				
** ≥ 4.00**	Referent	–	–	16.29 ± 5.58%
** 3.00–3.99**	1.08	0.36	0.0026	36.45 ± 6.47%
** < 3.00**	2.01	0.61	0.0011	59.27 ± 14.16%
**Race**				
** White**	Referent	–	–	21.98 ± 6.41%
** Racially Minoritized**	1.32	0.28	< 0.0001	51.37 ± 8.38%
**Times Enrolled in ANEQ 101**				
** First-Time Enrollment**	Referent	–	–	20.06 ± 3.81%
** Not First-Time Enrollment**	1.55	0.52	0.0032	54.25 ± 13.47%

No significant interactions were observed, so main effects are reported for indicating inadequacy in notetaking abilities (yes/no), when controlling for HSGPA, race, and times enrolled in the course.

The effects of attendance, both for lecture and laboratory periods, when accounting for potential confounders were analyzed using Models 7 and 8, respectively. The full outputs of these multivariate models are available in Supplementary Tables 3 and 4, available as supplementary data at *Translational Animal Science* online. Results of Model 7 show that when accounting for HSGPA, race, and times enrolled in the course, students that indicated attending lectures 90% of the time or less (missing 3 + lectures) had a significantly higher probability of earning a failing grade than those that indicated attending 100% or almost 100% of the time (missing 0–2 lectures) (80% to 90%: *p* = 0.0099; ≤ 70%: *p* = 0.0045). However, the students that reported attending almost all of the lectures and those that reported attending all of the lectures did not have different likelihoods of failing (*p* = 0.1854). It was determined that when controlling for HSGPA, race, and times enrolled, students attending 100% of lectures had a predicted 14.6% probability of failing and those attending almost 100% of lectures had a predicted 24.2% probability of failing, which is lower than the predicted 35.5% and 48.3% probabilities of failing for those attending 80% to 90% and 70% or less of lectures, respectively. A nearly identical pattern was observed when analyzing the association between laboratory period attendance and a student’s final grade in the course, where those that attended ≤ 70% of laboratory periods (missing 4 + laboratory periods) had a higher probability of failing than those that attended all or nearly all of the laboratory periods (*p* = 0.0076). However, there was no difference in probability of failing between those that attended 100% of the laboratory periods and those that attended 80% to 90% of the laboratory periods (*p* = 0.0950). Students that attended the laboratory periods 100% of the time had a predicted 23.24% probability of failing and students that attended the laboratory periods 80% to 90% of the time had a predicted 33.4% probability of failing, which are both much lower than the 57.5% predicted probability of failing for those that only attended the laboratory periods ≤ 70% of the time, among students with the same HSGPA, race, and number of times enrolled in the course. Therefore, students with poorer attendance patterns in the three-times weekly lectures and/or in the weekly laboratory periods had a greater likelihood of failing compared to those that attended more regularly, among those with similar demographic identities.

Lastly, all demographic factors determined to be associated with final grade in a bivariate analysis were tested together in one multivariable model (Model 9) to determine the effects of each factor on students’ final course grades in ANEQ 101, when accounting for the effects of the other demographic factors. There was a significant relationship between final grade and a student’s HSGPA, number of times enrolled, and their race, when accounting for the effects of the other two (*p* < 0.001; Table 8). There was also a significant interaction seen between first-generation (FG) status and HSGPA (*p* = 0.0397), so the final model output for Model 9 includes the main effects for race and times enrolled, as well as the interaction effect for HSGPA*FG status (Table 2). The results of this model indicate that the effect of FG status on final grade differs by HSGPA range, where among only students with a HSGPA of 4.00 or higher, FG students had a much higher predicted probability of failing than non-FG students, but, the same was not true for students with HSGPA 3.00–3.99 or for those with HSGPA less than a 3.00 (Table 8). Instead, minimal difference was seen in the likelihood of failing between FG and non-FG students in the 3.00–3.99 HSGPA range (31.1% and 31.5%, respectively) and for students in the less than 3.00 HSGPA range, FG students had a lower predicted probability of failing than non-FG students (39.8% and 62.9%, respectively; Table 8). The results of this multivariate model also indicate that students of any minoritized race have a 43.8% predicted probability of failing ANEQ 101, which is more than twice that of white students (18.0%), when controlling for HSGPA, FG status, and number of times enrolled (Table 8). Lastly, Model 9 results show that students that are retaking ANEQ 101 have approximately three times higher predicted probabilities of failing ANEQ 101 than those taking it for the first time (47.6% and 15.8%, respectively), when controlling for HSGPA, FG status, and race (Table 8). Overall, when controlling for the other demographic factors, FG students with a high HSGPA, non-FG students with a low HSGPA, racially minoritized students, and students that are retaking the course are more likely to fail ANEQ 101 than their respective counterparts.

**Table 8:** Multivariable analysis of the effects of a student’s demographic factors on their probability of earning a failing grade in ANEQ 101.

Predictor	Estimate	*SE*	*P* value	Predicted probability of failing ± *SE* (%)
High School GPA*First-Generation Status				
** 4.00 or higher**				
** First-Generation Student**	Referent	–	–	22.46 ± 13.79%
** Non-First-Generation Student**	−1.84	0.75	0.0158	4.41 ± 3.99%
** 3.00–3.99**				
** First-Generation Student**	Referent	–	–	31.08 ± 6.92%
** Non-First-Generation Student**	1.85	0.78	0.0184	31.51 ± 6.50%
** Less than 3.00**				
** First-Generation Student**	Referent	–	–	39.83 ± 21.23%
** Non-First-Generation Student**	2.77	1.31	0.0341	62.90 ± 16.87%
**Race**				
** White**	Referent	–	–	17.97 ± 5.52%
** Racially Minoritized**	1.27	0.29	< 0.0001	43.80 ± 8.23%
**Times Enrolled in ANEQ 101**				
** First-Time Enrollment**	Referent	–	–	15.83 ± 3.02%
** Not First-Time Enrollment**	1.57	0.51	0.0023	47.59 ± 13.41%

A significant interaction was observed between HSGPA and FG status (*p* = 0.0397), so the interaction term effects are reported for HSGPA*FG status, when controlling for race and times enrolled in the course. The main effects for HSGPA and FG status are not reported.

## Discussion

The College of Agricultural Sciences at Colorado State University (CSU) is currently experiencing enrollment numbers that are higher than ever, especially within the Department of Animal Sciences, yet 4-year graduation rates consistently hover in the middle to high 40% range. This shows that over half of enrolled students are not receiving and/or properly utilizing adequate support to succeed academically. While these numbers are specific to CSU (Colorado State University 2025a), the lack of effective student support strategies and limited awareness of those that are present are issues seen worldwide (Thomas et al. 2002; Johnson et al. 2022). This issue is further exacerbated by the shifting demographics and growing diversity on campuses, creating more opportunities for hidden obstacles to exist for students of all identities. Past studies have shown support strategies that are effective in improving student success in other STEM disciplines (Smith et al. 2005; Carvalho and West 2011; Anderson et al. 2021), but little is known about effective strategies for animal science students specifically. By more adequately supporting students, especially during their first semester, they are more likely to remain at the university and within the major (Noble et al. 2007; Permzadian and Credé 2016), which can lead to increased success later in their educational journeys as well as in their professional careers following graduation. Therefore, this study aimed to assist in these efforts by identifying factors associated with student success in the introductory animal science course at CSU, as well as demographic factors that could be impacting how current support structures influence the success of animal science students.

### Demographics

The current survey was distributed to all students that were enrolled in the introductory animal science course at CSU during Fall 2021, 2022, and 2023, with respondents primarily in their first year of a bachelor’s degree program in Animal Sciences. Introductory courses are typically taken by first-year students in a degree program or related fields, as they often serve as prerequisites for future required courses (Colorado State University n.d.; Peffer 2011; Lavoie 2019). Survey responses were collected from roughly 20% of all animal science students and nearly 100% of all first-year animal science students each semester. A majority of the respondents identified as white and as female, which aligns with both animal science specific demographics and university-wide demographics for CSU (Colorado State University 2022; Institutional Research Planning and Effectiveness and Colorado State University 2024). This also aligns with similar studies of animal science undergraduates conducted at other agricultural colleges and universities (Bobeck et al. 2014; White et al. 2015; Lavoie 2019; Carlson et al. 2024). A significant number of respondents were first-generation (FG) college students (31%), though they did not represent the majority. In the last decade, roughly a third of animal sciences students and nearly a quarter of all students at CSU identified as FG students (Institutional Research Planning and Effectiveness and Colorado State University 2024). According to a national report, 54% of all undergraduates and 48% of undergraduates at public 4-year universities identified as FG students as of 2020, with FG being defined as having parents who did not receive at least a bachelor’s degree (RTI International 2023). Therefore, the current study demographics align well with what is seen nationwide. The current study population also has a significant proportion of students that are transfer students, coming to CSU either from a different 2-year or 4-year university. According to the National Student Clearinghouse (2024), roughly 13.2% of all undergraduate students at 4-year universities are transfer students, which aligns with the 12.9% of students that identified as transfer students in the current study (National Student Clearinghouse 2024). Lastly, a larger percentage of respondents in the current study are not residents of Colorado (59%) compared to the 40% non-resident average across all degree programs at CSU. This is likely due to the current study’s respondent pool being primarily composed of animal science students, most of which indicated an interest in veterinary medicine following graduation. In 2023, the Doctor of Veterinary Medicine program at CSU was ranked #2 in the nation by the U.S. News and World Report (US News 2025), which brings in students from across the country that are interested in veterinary medicine.

### Academic behaviors associated with student success

According to data from the current survey, students that were involved in extracurricular activities within the department had lower probability of earning a failing grade in the Introductory Animal Science course at CSU. The specific types of involvement associated with final grade included departmental involvement in research, extracurricular communication with departmental faculty, and joining a club on campus. The proportion of students in the current survey that were involved in research (7%) is smaller than the university wide average of 15% (Colorado State University 2025b), but this is to be expected as only 6% of first-year students participated in research at CSU, compared to 30% by their fourth year (Dominguez and Novak 2019), and the large majority of current study respondents (75%) were in their first year of their degree program. Similarly, only 23% of survey respondents reported connecting with faculty, which is slightly lower than data from the National Survey of Student Engagement (NSSE), where 30% to 46% of CSU students and 21% to 39% of students at other large land-grant institutions reported at least one interaction with faculty outside class (Dominguez and Novak 2019). Further, the NSSE reported proportion of students who met with faculty outside of class time by topic they discussed with the faculty member, so the overall number of students that connected one-on-one with faculty could be even higher than what was reported. The discrepancy in findings between the current study and the NSSE data could be a reflection of the fact that the proportion of students that seek out intentional and meaningful faculty contact at other universities have been reported to fluctuate widely based on factors such as size and selectivity of the institution, student participation in research or pre-professional clubs, student perceptions of faculty members’ attitudes towards students, and an interaction of student race and university location (Fischer 2007; Hurtado et al. 2011). Lastly, a much larger proportion of respondents in the current survey indicated being in a club compared to the statistics reported by Colorado University as a whole, as well as by other universities (Hawkins 2015; Carlson et al. 2024; Colorado State University 2025b). This is to be expected due to the high percentage of respondents in the current survey that have an interest in applying for veterinary school following completion of a bachelor’s degree. The American Association of Veterinary Medical Colleges recommends students interested in applying for veterinary schools should join clubs related to animal science and/or pre-veterinary medicine and gain as much hands-on experience with animals as possible during their undergraduate experience in order to make their applications stronger and more competitive (American Association of Veterinary Medical Colleges 2025). Therefore, a population with a high concentration of students planning to apply for veterinary school is likely to have a high proportion of respondents who indicate club involvement.

The associations between final course grade and student involvement in the department from the current study align with results of previous studies conducted on undergraduates enrolled in science-based degree programs in universities across the United States. Sell et al. (2018) determined that involvement in undergraduate research was positively correlated with undergraduate GPA (Sell et al. 2018). Further, Hurtado et al. (2011) determined that being involved in research and/or clubs on campus is correlated to students interacting more with faculty members, and Komarraju et al. (2010) determined interactions with faculty are associated with improved academic performance. Thus, it can be concluded that engagement and involvement of undergraduate students have significant impacts on their grades. A student’s level of engagement and involvement, both within a specific class and within the larger department or college, is often related to their sense of belonging within their university, college, and/or department (Wilson et al. 2015; Strayhorn 2022). However, a student’s sense of belonging and willingness to get involved are also often influenced by their demographic identities. For example, Hurtado et al. (2011) found that African American students at Historically Black Colleges and Universities reported higher engagement, better academic performance, and stronger relationships with professors than their peers at predominantly white institutions due to their enhanced feeling of belonging in that community. Further research is needed to fully understand the relationship between demographic factors, a student’s sense of belonging, extracurricular involvement, and overall student success. Regardless of demographic factors, though, it has been reported that sense of belonging is influenced by positive interactions with faculty members and increased participation in extracurricular activities (Cwik and Singh 2022; Strayhorn 2022). Thus, faculty can support undergraduate success by encouraging departmental involvement, fostering inclusive professional learning communities, and building classroom- and department-wide environments that promote belonging for students of all identities and interests, particularly within their first year.

One specific form of involvement in the current study that was associated with reduced probability of failing was involvement in the daily care of department-owned dairy calves used for course activities and laboratory research. This aligns with a survey of undergraduate engineering students that describes a positive correlation between voluntary participation/involvement in additional hands-on learning opportunities and students’ self-efficacy in engineering principles and practices, which is associated with overall academic achievement (Bartimote-Aufflick et al. 2016; Morocz et al. 2016). Numerous studies have shown the positive impacts of hands-on and experiential learning, including but not limited to increased comprehension and retention of practical/technical skills (Smith et al. 2005; Bradberry and De Maio 2018; Robinson 2023), improvement of strategic and critical thinking skills (Robinson 2023), and increased student interest and motivation to participate and engage in learning (Reiling et al. 2003; Smith et al. 2005; Sullivan et al. 2023). It is likely due to these benefits, as well as numerous others, that hands-on on experiential learning opportunities have been seen to further increase a student’s self-efficacy in their chosen discipline (Anderson et al. 2021). Although all students in the study had access to hands-on learning in lab sections, only about one-third volunteered for additional experiences such as feeding, monitoring, and medicating calves. Low participation may stem from barriers like transportation, work commitments, lack of confidence with livestock, or other systemic factors. While such barriers cannot be eliminated entirely, they can be reduced by designing calf care opportunities with flexible time commitments, accessible transportation support, guided skill-building, and intentional efforts to create a supportive environment for novice students. Limiting these barriers and their effects on students may increase student involvement in extracurricular opportunities like calf care, which in this study was linked to lower predicted probabilities of failing ANEQ 101. Overall, offering opportunities for hands-on and experiential learning for first-year animal science students and continuously encouraging students to be involved in those opportunities throughout their academic careers can help improve their odds of succeeding academically in their chosen degree fields.

Additional academic behaviors associated with lower probabilities of failing ANEQ 101 included positive perceptions of self-adequacy in notetaking and study habits, greater perceived ease of studying for quizzes and exams, and positive attendance. However, no association was seen between the actual notetaking strategies or time spent studying and probabilities of failing. Much of recent research has highlighted the importance of good study skills and proper notetaking habits for student success, but few have defined the level of study skills by perceptions of adequacy on the part of the student. For example, a study by Sebesta and Speth (2017) reported improved examination scores for first-year college students in an introductory biology class for students that reported using self-regulated cognitive learning strategies taught in class to study, whereas those that did not utilize strong study skills did not improve on examination scores. However, the association was only seen between students performing certain study skills, without considering how students perceived the effectiveness of those skills. Further, a study by Mendezabal (2013) showed poor study habits among fourth-year college students, as defined by the Survey of Student Habits and Attitudes developed Holtzman et al. (1954), led to poorer scores on board examinations. While the survey does ask about teacher approval and educational acceptance of study strategies used, it still doesn’t factor in the component of how students feel about the adequacy of those skills for their own learning. For most students, it is likely those that have inadequate skills objectively are aware that this is the case, but there may be some students that utilize adequate techniques yet still feel their skills need improving to fit their own needs. Teaching self-regulated learning strategies encourages students to think critically, connect new material to prior experiences, and apply knowledge to real-world contexts rather than relying on rote memorization (Nilson 2023). Instructors who explicitly teach how to learn and study effectively for specific courses can improve students’ confidence in their study and notetaking abilities (McGuire 2023), which in turn could help improve their odds of passing. However, societal barriers like the need to work while in school, which disproportionately affect first-generation and lower socioeconomic students (Pascarella et al. 2004; Green 2021; Sokin 2021), can limit how effectively these strategies enhance student success, underscoring the need for equitable instructional approaches. This aligns with current study findings as well, which showed first-generation and students identifying as a non-minority race, which more often come from lower socioeconomic backgrounds compared to white-identifying students (American Psychological Association 2017), had higher odds of failing compared to non-first-generation students and white-identifying students, respectively. Collectively, current findings paired with previous research show that students’ perceptions of their own study abilities, combined with structural barriers that limit access to learning opportunities, play a critical role in academic success and should be central considerations when designing equitable support strategies.

Lastly, better attendance in both lecture and laboratory periods were associated with higher odds of passing ANEQ 101 even though no grades were given specifically for attendance, which is corroborated by past studies showing positive correlations between attendance in a class and a student’s final grade in that class (Halpern 2007; McMillan et al. 2009; Khan 2022). Student attendance, or lack thereof, is often impacted by social factors and personal choice on the part of the student (Sloan et al. 2020). Faculty have long advocated for improved attendance, and prior research shows that students themselves recognize the academic benefits of attending lectures (Alamoudi et al. 2021). Despite this, many students still choose not to attend for a variety of reasons—including early class times, the convenience of recorded lectures, conflicting responsibilities like exam preparation or work, and dissatisfaction with instructional quality. These decisions are often influenced by factors such as gender, GPA, and academic standing (Alamoudi et al. 2021). Since many of these influences are unlikely to change, further research is needed to explore effective strategies for promoting attendance and to assess whether course content should be adapted to better support students who cannot attend in person. Ultimately, understanding and addressing these barriers is essential for creating more inclusive and flexible learning environments.

In short, it has been seen that departmental involvement, adequate and personalized learning strategies, and regular attendance can all increase a student’s probability of passing a course. However, many of these opportunities are hidden behind societal and systemic barriers associated with student demographics and background characteristics that make them less accessible for all students.

### Demographic factors associated with student success

The second objective of the current study was to identify personal demographic and/or background characteristics of students that are associated with final course grade, regardless of a student’s academic behaviors. Current results indicate that students retaking the course, those that are of a racially minoritized identity, and FG students with a HSGPA ≥ 4.00 have a higher probability of failing, compared to first-time enrollees, those identifying their race as white, and non-FG students with a HSGPA ≥ 4.00, respectively. The results regarding students retaking the course align with a study conducted on students enrolled in an introductory economics class in Canada that showed lower grades among students who had previously failed or withdrawn from the course compared to those of students taking the course for the first time (Armstrong and Biktimirov 2014). However, the current study results regarding the effects of race are contradicted by past research. Only a small proportion of papers have explicitly explored the impact of race or ethnicity on student success matrices overall, and of those that have been done, no significant correlations have been reported between race or ethnicity and course grades, self-efficacy, or overall academic success in post-secondary education (Hackett et al. 1992; Lavoie 2019). The fact that the current study found significant differences where past studies did not could be due to the large overall sample size and/or to the fact that the current study had a higher proportion of racially minoritized students compared to the previous literature examining the impaLavoie 2019ct of race specifically within animal science. For example, in one recent study conducted at University of Nebraska-Lincoln by Lavoie (2019), the study population consisted of only 193 students, 93% of which identified their ethnicity as Caucasian (Lavoie 2019). The lack of variation in ethnicity in the study population in the Lavoie et al. paper could be why that study did not see an association between ethnicity and student final grade, student motivation, or self-efficacy. Lastly, numerous studies support the strength of HSGPA (Wesley 1994; Geiser and Santelices 2007; Kurlaender and Cohen 2019) as a predictor of academic success in higher education, but none have explored the interaction between FG status and HSGPA in terms of overall success. However, one previous study does describe FG status as a strong predictor of HSGPA, which begins to illuminate the interconnectedness of the two demographic factors (Strayhorn 2007). While results from the present study indicate that FG status among students with high HSGPA, student race, and number of times enrolled in the course are all related to students’ final grade, further research is needed to understand the complexities of the relationships between demographic factors and how they impact student success.

By more specifically detailing the relationship between multiple demographic factors, as well as how they relate to a student’s willingness and/or ability to perform certain academic behaviors, faculty can begin to tailor current support strategies for animal science students to increase equitability and effectiveness of these efforts. Current methods of supporting students identifying with certain demographic characteristics known to put them at an increased likelihood of failing vary by institution and demographic of interest. At CSU, students with poor high school performance are flagged as “Students Recommended for Support (SRS)” during admissions (Colorado State University 2021) and all students are assigned a Support Priority Level each semester, with “High Priority” students intended to receive more advisor guidance (CSU Navigate 2024). However, CSU’s persistently low graduation rates suggest these systems do not fully meet student needs, potentially due to the influence of demographic factors on how support is experienced. For example, best practices at Historically Black Colleges and Universities (HBCUs) and Hispanic-Serving Institutions (HSIs) to support students of minoritized ethnic and/or racial identities include, but are not limited to, promoting inclusive faculty and student interactions, celebrating cultural identity and representation, providing multilingual academic support, and fostering the success of minoritized faculty members, all aimed at enhancing students’ sense of belonging and satisfaction in their degree programs (Baker 2013; Valentin 2018; Garcia 2023; Baldwin and O’Sullivan 2025). Improved sense of belonging, community, and satisfaction in a college or degree program have been shown to improve academic success and graduation rates for all students, but especially those of minoritized identities (Suhre et al. 2007; Sinclaire 2014; Baldwin and O’Sullivan 2025). Further, all students, regardless of their identities, are more likely to be involved on campus and engaged in their coursework when they connect one-on-one with faculty members that are of a similar identity to themselves and/or are provided with opportunities to support or contribute to a community group they identify with (Palmer et al. 2011; Baldwin and O’Sullivan 2025). However, according to recent reports by the National Center for Education Statistics, more than 70% of university faculty members nationwide are white (NCES 2022), and only 15% of faculty at Colorado State University identify as an underrepresented race or ethnicity (Colorado State University Faculty Success 2024). This limits the ability for students of minoritized identities to connect with mentors of similar identities and creates a barrier for belonging. Further, little is reported about other minoritized identities outside of race or ethnicity and how prevalent they are among faculty, nor about how the lack of representation might impact a student’s sense of belonging on campus. Realistically, however, perfectly aligning faculty demographics to those of the students is nearly impossible, so further research needs to be conducted to explore ways to help students make meaningful connections on campus regardless of faculty demographics. In conclusion, demographic and background factors such as race, HSGPA, FG status, and times enrolled in a course do create systemic barriers to student success in higher education, but further research needs to be done to further define this impact and explore solutions to overcoming these barriers.

## Conclusion

Overall, the success of first-year animal science students in an introductory food animal science course at CSU can be influenced by numerous factors, including their involvement in extracurricular activities, their propensity to connect one-on-one with faculty members, regularity of their attendance, and their perceptions of adequacy in the quality of their notetaking and study abilities. Additionally, student demographic and background characteristics such as race, high school GPA, first-generation status, and number of times enrolled in a course are directly associated with their final grades, where students of a minoritized race, those with high school GPA less than 4.0, first-generation students, and those repeating the course are more likely to fail. These demographic factors are also known to be indirectly related to course grade by affecting how the previously mentioned involvement, engagement, attendance, and learning habit factors impact course grade. To effectively support students in their first year, animal science programs should implement holistic approaches that combine academic interventions that promote course engagement, regular attendance, help students build individualized and effective learning strategies, create more culturally responsive support, and encourage more meaningful student-faculty interactions early on each semester. One way this could be achieved is through meaningful first-year student seminars, such as those described in Permzadian and Credé (2016), that are designed around these specific goals. Future studies should be conducted to more explicitly define how academic behaviors and demographics impact other student success matrices, as well as promote contemporary teaching strategies that meet the needs of more diverse learners in animal science programs.

## Supplementary Material

txag091_Supplementary_Data
