# Supplementary material for: Factors associated with student success in an introductory level animal science course
Source: Transl Anim Sci. 2026 Jul 2;10:txag091. doi: 10.1093/tas/txag091 (PMC13350890; doi:10.1093/tas/txag091)
Supplement: txag091_Supplementary_Data [file txag091_supplementary_data.docx]

# Supplemental Materials

Supplementary Table 1: Survey questions included in analysis and their analysis categories.

| Outcome of Interest |
| --- |
| What letter grade do you have currently in ANEQ 101? *^3^* |
| Predictor of Interest - Involvement |
| Have you reached out to and/or connected 1-on-1 with faculty? *^1^* |
| Are you involved in any research activities in the Department of Animal Sciences? *^1^* |
| Did you volunteer to help feed the dairy calves during the first 7 weeks of the semester? *^1^* |
| I have joined a student club or organization at CSU. *^2^* |
| Predictor of Interest – Notetaking |
| How have you been taking notes in ANEQ 101? *^3^* |
| The quality of my notetaking was inadequate. *^2^* |
| I need to improve my notetaking in courses like ANEQ 101. *^2^* |
| Predictor of Interest – Study Habits |
| How much time per week did you study/prepare for weekly quizzes in ANEQ 101? *^4^* |
| How much time per week did you study/prepare for the exams in ANEQ 101? *^4^* |
| I did not study as much as I should have. *^2^* |
| It was difficult for me to study/prepare for the quizzes and exams. *^2^* |
| I need to improve my study skills and habits in general. *^2^* |
| Predictor of Interest – Attendance |
| Approximately what percentage of lectures did you attend this fall in ANEQ 101? *^3^* |
| Approximately what percentage of laboratory periods did you attend this fall in ANEQ 101? *^3^* |
| Predictor of Interest – Demographics |
| Is this semester the first time you have ever enrolled in ANEQ 101 at CSU? *^1^* |
| At the start of the semester, what was your major at CSU? *^4^* |
| Currently, what is your major at CSU? *^4^* |
| What year are you currently at CSU? *^3^* |
| What is your gender? *^3^* |
| How would you best describe your race? *^3^* |
| What was your GPA in high school, on a 4.0 scale? *^3^* |
| I am enrolled in the honors program at CSU. *^1^* |
| I am the first generation in my family to attend college. *^1^* |
| I am an in-state student. *^1^* |
| I am a transfer student (and this is my first semester at CSU). *^1^* |
| I am interested in going to veterinary school. *^1^* |
| *^1^* Dichotomous measure *^2^* Likert scale measure *^3^* Multiple choice measure *^4^* Free response measure |

Supplementary Table 2: Multivariable analysis of the effects of a perceived need to improve one’s own notetaking strategies on that student’s probability of earning a failing grade in ANEQ 101. No significant interactions were observed, so main effects are reported for perceived need to improve notetaking (yes/no), when controlling for HSGPA, race, and times enrolled in the course.

| Predictor | Estimate | SE | *P*-value | Predicted Probability of Failing ± SE (%) |
| --- | --- | --- | --- | --- |
| Perceived Need to Improve Notetaking |  |  |  |  |
| Yes | Referent | - | - | 35.57 ± 7.46% |
| No | -1.56 | 0.45 | 0.0006 | 10.38 ± 4.81% |
| High School GPA |  |  |  |  |
| ≥ 4.00 | Referent | - | - | 8.81 ± 3.57% |
| 3.00-3.99 | 1.11 | 0.37 | 0.0031 | 22.71 ± 5.68% |
| < 3.00 | 1.78 | 0.62 | 0.0046 | 36.29 ± 14.27% |
| Race |  |  |  |  |
| White | Referent | - | - | 12.23 ± 4.37% |
| Racially Minoritized | 1.19 | 0.28 | < 0.0001 | 31.45 ± 8.22% |
| Times Enrolled in ANEQ 101 |  |  |  |  |
| First-Time Enrollment | Referent | - | - | 10.00 ± 2.56% |
| Not First-Time Enrollment | 1.64 | 0.53 | 0.0022 | 36.51 ± 13.38% |

Supplementary Table 3: Multivariable analysis of the effects of lecture attendance on their probability of earning a failing grade in ANEQ 101. No significant interactions were observed, so the main effects are reported for lecture attendance when controlling for HSGPA, race, and times enrolled in the course.

| Predictor | Estimate | SE | *P*-value | Predicted Probability of Failing ± SE (%) |
| --- | --- | --- | --- | --- |
| Lecture Attendance |  |  |  |  |
| 100% (Missed 0 Classes) | Referent | - | - | 14.58 ± 6.38% |
| Almost 100% (Missed 1-2 Classes) | 0.62 | 0.47 | 0.1954 | 24.19 ± 6.94% |
| 80-90% (Missed 3-10 Classes) | 1.17 | 0.45 | 0.0099 | 35.46 ± 8.21% |
| < 70% (Missed ≥ 11 Classes) | 1.70 | 0.60 | 0.0045 | 48.30 ± 11.63% |
| High School GPA |  |  |  |  |
| ≥ 4.00 | Referent | - | - | 12.43 ± 4.45% |
| 3.00-3.99 | 1.13 | 0.36 | 0.0017 | 30.59 ± 5.91% |
| < 3.00 | 2.04 | 0.60 | 0.0007 | 52.23 ± 14.49% |
| Race |  |  |  |  |
| White | Referent | - | - | 17.51 ± 5.32% |
| Racially Minoritized | 1.31 | 0.28 | < 0.0001 | 44.06 ± 8.14% |
| Times Enrolled in ANEQ 101 |  |  |  |  |
| First-Time Enrollment | Referent | - | - | 17.16 ± 3.27% |
| Not First-Time Enrollment | 1.36 | 0.54 | 0.0113 | 44.66 ± 13.47% |

Supplementary Table 4: Multivariable analysis of the effects of laboratory period attendance on their probability of earning a failing grade in ANEQ 101. No significant interactions were observed, so the main effects are reported for laboratory attendance when controlling for HSGPA, race, and times enrolled in the course.

| Predictor | Estimate | SE | *P*-value | Predicted Probability of Failing ± SE (%) |
| --- | --- | --- | --- | --- |
| Laboratory Period Attendance |  |  |  |  |
| 100% (Missed 0 Laboratory Periods) | Referent | - | - | 23.24 ± 7.27% |
| 80-90% (Missed 1-3 Laboratory Periods) | 0.50 | 0.30 | 0.0950 | 33.40 ± 7.45% |
| < 70% (Missed ≥ 4 Laboratory Periods) | 1.50 | 0.56 | 0.0076 | 57.53 ± 13.84% |
| High School GPA |  |  |  |  |
| ≥ 4.00 | Referent | - | - | 16.58 ± 5.90% |
| 3.00-3.99 | 1.20 | 0.36 | 0.0010 | 39.67 ± 7.43% |
| < 3.00 | 2.07 | 0.62 | 0.0008 | 61.14 ± 14.31% |
| Race |  |  |  |  |
| White | Referent | - | - | 23.79 ± 7.07% |
| Racially Minoritized | 1.31 | 0.28 | < 0.0001 | 52.74 ± 9.09% |
| Times Enrolled in ANEQ 101 |  |  |  |  |
| First-Time Enrollment | Referent | - | - | 20.72 ± 4.13% |
| Not First-Time Enrollment | 1.63 | 0.55 | 0.0029 | 57.14 ± 14.14% |

Supplementary Table 5: Bivariate analysis between significant demographic factors and each predictor of interest significantly associated with a student’s probability of earning a failing grade in ANEQ 101. *P*-values are reported for each combination of predictors, with **bolded** terms indicating significant association (*P* < 0.05).

| Predictor | *P*-value | | | |
| --- | --- | --- | --- | --- |
|  | Times Enrolled | First-Generation Status | HSGPA | Race |
| Involvement |  |  |  |  |
| Involvement (Combined) | 0.3460 | 0.0850 | **< 0.0001** | **0.0217** |
| Connecting 1-on-1 with Faculty | 0.5769 | 0.5425 | **0.0046** | 0.5481 |
| Involvement in Department Research | 0.2429 | 0.1836 | **0.0009** | 0.1894 |
| Volunteering to Feed Laboratory Calves | 0.3715 | 0.4524 | **0.0416** | **0.0159** |
| Involvement in a Student Club or Organization | 0.0713 | **0.0303** | **0.0002** | **0.0033** |
| Perceived Inadequacy of Notetaking |  |  |  |  |
| Perceived Inadequacy in Personal Notetaking   Ability | **0.0187** | **0.0253** | 0.2501 | **0.0102** |
| Perceived Need for Improved Notetaking | 0.1633 | **< 0.0001** | **< 0.0001** | **< 0.0001** |
| Perceived Inadequacy of Study Habits |  |  |  |  |
| Perceived Inadequacy in Time Spent Studying | 0.6538 | **0.0396** | 0.0501 | **0.0003** |
| Experienced Difficulty in Studying/Preparing   for Quizzes and Exams | **0.0121** | **0.0080** | 0.0832 | **< 0.0001** |
| Perceived Need for Improvement in Study   Skills/Habits | 0.4942 | **0.0009** | **0.0002** | **0.0002** |
| Attendance |  |  |  |  |
| Lecture Attendance | **< 0.0001** | 0.4211 | **0.0022** | 0.2649 |
| Lab Attendance | **0.0020** | 0.4407 | **0.0086** | 0.1508 |
| Demographics |  |  |  |  |
| Times Enrolled | **-** | 0.0518 | 0.1442 | **0.0001** |
| First-Generation Status | 0.0518 | **-** | **0.0135** | **< 0.0001** |
| HSGPA | 0.1442 | **0.0135** | **-** | 0.0763 |
| Race | **0.0001** | **< 0.0001** | 0.0763 | **-** |

Supplementary Questionaire: Copy of the 2023 Student Evaluation and Feedback student survey used for data collection that was distributed to all students enrolled in ANEQ 101.

Background: The goal of this **anonymous** survey is to improve the quality of instruction provided in ANEQ 101 by acquiring feedback from students related to their experiences in, and views about, this course. There are no “right” or “wrong” answers to these questions. Ultimately, we hope to address barriers to success in ANEQ 101, primarily among new first-year and transfer students. Please feel free to add comments to clarify your responses.

**SECTION 1 – COURSE OVERVIEW**

1. Is this semester the first time you have ever been enrolled in ANEQ 101 at CSU?

1. Yes
2. No

2. How would you rate the quality and value of information you were provided in ANEQ 101?

1. Very high quality
2. High quality
3. Average
4. Low quality
5. Very low quality

Comments:

 3. Did ANEQ 101 meet your expectations?

1. Yes
2. No

If no, what was the primary reason why not?

 4. What did you think about having other faculty members from the Department of Animal Sciences guest lecture in ANEQ 101?

1. Even more guest lectures should be included from faculty, including on all topic areas
2. The number of faculty members that provided guest lectures was appropriate
3. Fewer faculty members should provide guest lectures
4. It would be best to have 1 main instructor provide all lecture material

Comments:

5. Of the following faculty member(s) who guest lectured, indicate which you enjoyed the **most**with an “**M**” (maximum of 2) and which you enjoyed the **least** with an “**L**” (maximum of 2)?

__ Lily Edwards-Callaway (Animal Ethics/Welfare)

__ Mahesh Nair (Meat Science)

__ Shawn Archibeque (Nutrition)

__ Noa Roman-Muniz (Lactation)

__ Temple Grandin (Animal Handling)

__ Libby Bigler (Beef Quality Assurance)

__ Pablo Pinedo (Reproduction)

__ Caitlin Cadaret (Reproduction)

__ Stephen Coleman (Genetics)

__ Scott Speidel (Animal Breeding)

__ Jennifer Martin (Meat Palatability - Lab)

__ Mesa Kutz (Livestock Judging - Lab)

__ Catie Cramer (Calf Health, Dairy Production)

__ Haley Cole (Meat Judging - Lab)

__ Jessica Metcalf (Microbiome)

6. This fall, have you reached out to and/or connected 1-on-1 with a faculty member who guest lectured in ANEQ 101?

1. Yes
2. No

If yes, who was the faculty member?

7. Was it valuable to have lecture notes (i.e., printed slides) handed out before each lecture?

1. Yes
2. No

Comments:

 8. Should topics about companion animals, wildlife, and/or exotic species be included in ANEQ 101 or another introductory course, or should ANEQ 101 maintain its focus on food animals?

1. Yes, include other topics
2. No, maintain food animal focus in ANEQ 101

9. Please provide any additional comments related to the **lecture** portion of ANEQ 101:

**SECTION 2 – COURSE PERFORMANCE**

10. Prior to the first day of class, what letter grade did you think you would achieve in ANEQ 101 this fall?

1. A
2. B
3. C
4. D
5. F

11. Approximately, what letter grade do you have currently in ANEQ 101?

1. A
2. B
3. C
4. D
5. F

12. What final course letter grade are you likely to receive in ANEQ 101 at the end of this semester?

1. A
2. B
3. C
4. D
5. F

13. Approximately how much time per week did you study/prepare for the weekly **quizzes** in ANEQ 101 this semester?

___ minutes (or___ hours) per quiz

14. Approximately how much time per exam did you study/prepare for the 2 **exams** in ANEQ 101 this semester?

___ minutes (or___ hours) per exam

15. Were you also enrolled in ANEQ 180A2 Exploring Student Success (1 credit) this semester?

1. Yes
2. No

16. How have you been taking notes in ANEQ 101 this fall?

1. I don’t really take notes, but I listen and follow along with the slides instead
2. I write a few notes on the printed slides that are handed out
3. I write a lot of notes on the printed slides that are handed out
4. I take a lot of notes separately in a notebook and not on the printed slides
5. I take notes on a tablet or other electronic device that has the slides on it
6. I take notes on a blank tablet or other electronic device (with no slides on it)
7. Other: _______________________

17. Approximately what percent of the 3-times-a-week **lectures** (M, W, F at 10:00-10:50 am) did you attend this fall in ANEQ 101? (mark only one)

1. 100% (I didn’t miss a single lecture)
2. Almost 100% (I missed ~1-2 lectures)
3. ~90% (I missed ~3-5 lectures this semester)
4. ~80% (I missed ~6-10 lectures this semester – about 1 every other week)
5. ~70% (I missed ~11-15 lectures this semester – about 1 per week)
6. ~60% (I missed ~16-19 lectures this semester – more than 1 per week)
7. ~50% or less (I missed ~20-25 or more lectures this semester – up to 2 per week)

18. On days that you did NOT attend **lecture**, what was the primary reason?

19. What was your GPA (grade point average) in high school, on a 4.0 scale (i.e., out of 4.0)?

1. Greater than a 4.0 (this can occur in some Colorado high schools)
2. Very close to a 4.0
3. 3.5 to 3.9
4. 3.0 to 3.4
5. 2.5 to 2.9
6. 2.0 to 2.4
7. Below a 2.0

20. Are you involved in any research activities in the Department of Animal Sciences?

1. Yes
2. No

If yes, was this involvement a result of your enrollment in ANEQ 101?

21. What are some main reasons that you may not do as well in this course as you had originally hoped? (**mark all that apply**)

1. I am getting the grade I had hoped for
2. Course material was too difficult to comprehend
3. The amount of material was overwhelming
4. Not enough review sessions or help sessions were provided
5. I did not study as much as I should have
6. I did not attend lectures or labs as much as I should have
7. I missed too many quizzes
8. I did not turn in at least 1 homework assignment
9. The quality of my notetaking was inadequate
10. It was difficult for me to study/prepare for the quizzes and exams
11. I did not take this course seriously
12. Other: _________________________

Comments:

22. When considering obstacles or challenges that may have prevented you from being as successful in ANEQ 101 as you wanted, please provide your level of agreement with each of the following statements: *(SA = Strongly Agree, A = Agree, D = Disagree, SD = Strongly Disagree)*

Financial challenges were an obstacle to my success  SA    A    D    SD

Legal challenges were an obstacle to my success  SA    A    D    SD

Family responsibilities were an obstacle to my success  SA    A    D    SD

Mental health challenges were an obstacle to my success  SA    A    D    SD

Physical health challenges were an obstacle to my success  SA    A    D    SD

Learning challenges were an obstacle to my success  SA    A    D    SD

Work responsibilities were an obstacle to my success  SA    A    D    SD

Something else was an obstacle to my success   SA    A    D    SD

**SECTION 3 – WEEKLY LABS**

23. Who was the **lab instructor** for your face-to-face weekly **lab** section?

1. Jason Ahola (Mon 12:00-1:50 pm)
2. Lexie Miller (Mon 2:00-3:50 pm)
3. Tyler Thomas (Tues 11:00-12:50 pm)
4. Abbey Schiefelbein (Tues 3:00-4:50 pm)
5. Rachael Stucke (Wed 1:00-2:50 pm)
6. Claire Okoren (Wed 3:00-4:50 pm)
7. Erica Machuca (Thur 11:00-12:50 pm)
8. Paxton Sullivan (Thur 3:00-4:50 pm)

24. Approximately what percent of ANEQ 101 once-a-week **labs** did you attend this fall?

1. 100% (I didn’t miss a single lab)
2. ~90% (I missed 1-2 labs all semester)
3. ~80% (I missed ~3 labs this semester)
4. ~70% (I missed ~4-5 lectures this semester)
5. ~60% (I missed ~6 labs this semester)
6. ~50% or less (I missed ~7 or more labs this semester)

25. On days you did NOT attend **lab**, what was the primary reason?

26. Of the following lab topics from this semester, indicate which you enjoyed the **MOST** with an “**M**” (maximum of 3) and which you enjoyed the **LEAST** with an “**L**” (maximum of 3)?

__ *Calf handling and anatomy (week 2)

__ *Calf health evaluation (week 4)

__ *Calf BQA procedures (week 5)

__ *Calf management/castration (week 6)

__ Digestive tract dissection (week 7)

__ Reproductive tract dissection (week 8)

__ Meat product tasting (week 9)

__ Livestock evaluation at ARDEC (week 10)

__ Sheep handling at ARDEC (week 11)

__ ARDEC facility tour (week 12)

__ Dairy product processing & tasting (week 13)

__ Meat evaluation and facility tour (week 14)

__ Quiz Bowl (week 15)

**These labs were held at foothills*

For the above lab topics that were your **MOST** desirable, why did you like them?

For the lab topics that were your **LEAST** desirable, why did you not like them?

 27. Did you volunteer to help feed the dairy calves during the first 7 weeks of the semester?

1. Yes
2. No

If yes, approximately how many total times did you help feed calves?

If you volunteered, please describe any value this experience provided to you:

28. Did using dairy calves in 4 labs at the beginning of the semester help with your learning?

1. Yes
2. No

If yes, please elaborate…

29. Should we include the dairy calf project in future labs in ANEQ 101?

1. Yes
2. No

Please elaborate on your answer…

 30. Are there specific aspects of the ANEQ 101 dairy calf project that should be changed?

1. Yes
2. No

If yes, please explain…

31. What topic(s) or activities would you suggest being added as a **lab session** in the future?

32. Relative to your **lab instructor**, please provide your level of agreement with each of the following statements: *(SA = Strongly Agree, A = Agree, D = Disagree, SD = Strongly Disagree)*

They were organized, prepared, and used lab time efficiently SA    A    D    SD

They explained topics clearly and was easy to understand SA    A    D    SD

They were accessible and helpful outside of lab time  SA    A    D    SD

They encouraged student participation during lab   SA    A    D    SD

They stimulated my interest in the topic   SA    A    D    SD

They cared about students, including them being successful  SA    A    D    SD

They created a welcoming and inclusive environment  SA    A    D    SD

33. In general, what did your **lab instructor** do well?

34. In what ways could your **lab instructor** improve as a teacher?

35. Please provide any additional feedback regarding the **lab**portion of this course:

**SECTION 4 – CHALLENGES**

36. Regarding ANEQ 101 and the Department of Animal Sciences, please provide your level of agreement with each of the following statements: *(SA = Strongly Agree, A = Agree, D = Disagree, SD = Strongly Disagree)*

I ask questions in ANEQ 101 **lecture**   SA    A    D    SD

I ask questions in ANEQ 101 **lab**   SA    A    D    SD

I take detailed notes during ANEQ 101 lectures and labs   SA    A    D    SD

I keep track of ANEQ 101 assignment due dates, quizzes, and

exams in a calendar SA    A    D    SD

I need to improve my notetaking in courses like ANEQ 101  SA    A    D    SD

37. Regarding ANEQ 101 and the Department of Animal Sciences, please provide your level of agreement with the following statements:*(SA = Strongly Agree, A = Agree, D = Disagree, SD = Strongly Disagree)*

I am satisfied with simply passing all my classes (i.e., C grade)  SA    A    D    SD

I need to improve my study skills and habits in general SA    A    D    SD

I need to accept greater responsibility for my actions (attending

class, studying, completing assignments) SA    A    D    SD

I need to improve how I manage my time  SA    A    D    SD

ANEQ 101 is an academically rigorous course  SA    A    D    SD

The Animal Science major is the right major for me  SA    A    D    SD

38. Regarding ANEQ 101 and the Department of Animal Sciences, please provide your level of agreement with the following statements: *(SA = Strongly Agree, A = Agree, D = Disagree, SD = Strongly Disagree)*

I have attended instructor and/or TA office hours for ANEQ 101 SA    A    D    SD

I have joined a student club or organization at CSU  SA    A    D    SD

I feel welcomed in the Department of Animal Sciences as I am  SA    A    D    SD

My instructors make me feel safe to participate in ANEQ 101  SA    A    D    SD

Other students make me feel safe to participate in ANEQ 101  SA    A    D    SD

I intend to return to CSU in the Spring semester  SA    A    D    SD

If you answered D or SD, what is the primary reason you may not return?

39. Has your experience in ANEQ 101 and the Department of Animal Sciences been what you expected it would be?

1. Yes
2. No

If no, please elaborate on your answer…

**SECTION 5 – DEMOGRAPHICS**

40. At the start of the fall semester, what was your major at CSU?

41. Currently, what is your major at CSU?

42. What year are you at CSU currently?

1. Freshman
2. Sophomore
3. Junior
4. Senior

43. What is your gender?

1. Female
2. Male
3. Transgender female
4. Transgender male
5. Gender variant / non-conforming
6. Not listed: _________________
7. Prefer not to answer

44. How would you best describe yourself?

1. White
2. Hispanic
3. American Indian or Alaska Native
4. Black or African American
5. Asian
6. Multiracial
7. Other: _____________

45. Please indicate your preference for meat consumption:

1. I am a meat eater
2. I am a vegetarian (I don’t eat meat, but I do consumer or use other animal products)
3. I am a vegan (I don’t consume or use any products from animals)
4. Other: __________________

46. Please respond to the following statements:

           Circle one:

I am enrolled in the honors program at CSU Yes / No

I am the first generation in my family to attend college Yes / No

I am an in-state student Yes / No

I am a transfer student (and this is my first semester at CSU)  Yes / No

I am living away from home for the first time this fall Yes / No

47. Please respond to the following statements:

           Circle one:

Before ANEQ 101, I had prior experience with food animal livestock  Yes / No

Before ANEQ 101, I had prior experience with non-food animal

 livestock (i.e., horses, alpacas, llamas, etc.)  Yes / No

My only animal experience is with companion animals (dogs, cats) Yes / No

Growing up, I participated in a livestock project in 4-H and/or FFA Yes / No

I am interested in going to veterinary school  Yes / No

I am interested in a career in the food animal production industry  Yes / No

I am interested in a career working with companion animals (dogs, cats)  Yes / No

I am interested in a career working with wildlife or exotic animals  Yes / No

During this fall semester I have considered changing my major at CSU  Yes / No

48. Do you currently work while attending CSU?

1. Yes
2. No

If yes, approximately how many hours per week do you work?

1. 1-10 hours / week
2. 11-20 hours / week
3. 21-30 hours / week
4. 31-40 hours / week
5. 41 or more hours / week

49. Is there anything else you would like to share about your experiences in ANEQ 101 this fall?
